# Sepsis Diagnostic Excellence: Development and Validation of a Theoretical Framework for Conceptualizing Accurate, Timely Sepsis Diagnosis

**DOI:** 10.1097/CCE.0000000000001490

**Published:** 2026-09-25

**Authors:** Sarah A. Birken, Sachita Shrestha, Laura S. Clark, Cheyenne R. Wagi, Kandice L. Reilly, Marc A. Kowalkowski, Jessica A. Palakshappa, Jessie E. King, Chadwick D. Miller, Stefanie S. Sebok-Syer, Stephanie P. Taylor

**Affiliations:** 1 Department of Implementation Science, Wake Forest University School of Medicine, Winston-Salem, NC.; 2 Division of Hospital Medicine, Department of Internal Medicine, University of Michigan, Ann Arbor, MI.; 3 Department of Social Sciences and Health Policy, Wake Forest University School of Medicine, Winston-Salem, NC.; 4 Department of Internal Medicine, Wake Forest University School of Medicine, Winston-Salem, NC.; 5 Department of Emergency Medicine, Wake Forest University School of Medicine, Winston-Salem, NC.; 6 Department of Emergency Medicine, Stanford University, Stanford, CA.; 7 Institute for Healthcare Policy and Innovation, University of Michigan, Ann Arbor, MI.

**Keywords:** clinical decision-making, diagnostic errors, emergency services, hospital, Sepsis

## Abstract

**OBJECTIVES:**

Develop and empirically validate the Sepsis Diagnostic Excellence (SepsisDx) Theoretical Framework of multilevel factors influencing accurate, timely sepsis diagnosis.

**DESIGN:**

Observational qualitative semi-structured interviews.

**SETTING AND PARTICIPANTS:**

Twenty hospitals spanning four states and two health systems within the United States were purposively selected to maximize location, system structure, and patient population representation.

**ANALYSIS:**

We conducted 41 interviews with key informants from 13 hospitals, including top managers (e.g., system leaders), mid-level managers (e.g., emergency department (ED) heads; sepsis coordinators), and patient-facing clinicians and staff (e.g., unit nurses). We developed an interview guide by integrating three extant frameworks that collectively conceptualize sepsis diagnosis as a behavior among healthcare providers that is influenced by factors at micro-, meso-, and macroorganization levels. To develop SepsisDx, we used template analysis to identify constructs that influenced accurate, timely sepsis diagnosis and rapid analysis to identify relationships among the framework’s constructs. We refined preliminary findings based on feedback from a study advisory board and interview participants.

**RESULTS:**

Interviews validated the central premise of SepsisDx—that is, sepsis diagnosis is influenced by the interaction of factors at micro-, meso-, and macrolevels. The most salient individual-level factor influencing timely sepsis diagnosis was attention (i.e., noticing, encoding, interpreting, and focusing time and effort). Attention was reduced by ambiguous clinical presentations and environmental shocks (e.g., COVID-19). Various strategies increased organization-level attention while decreasing provider-level attention (e.g., audit and feedback). Strategies’ effectiveness was moderated by hospital characteristics such as staffing.

**CONCLUSIONS:**

The SepsisDx framework clarifies *which* factors influence sepsis diagnosis and *how* these factors operate through specific mechanisms. Accurate, timely sepsis diagnosis depends on organizational attention that aligns with individual provider attention to potential sepsis diagnoses. Leaders may improve sepsis diagnosis by pursuing health system- and organization-level strategies that reflect individual-, team-, and emergency-department-level experiences.

**KEY POINTS**

- **Question:** Which multilevel factors influence accurate, timely sepsis diagnosis, and how?
- **Findings:** This observational qualitative semi-structured interview study of 41 key informants from 13 hospitals spanning four states and two health systems, purposively selected to maximize representation of location, system structures, and patient populations, revealed that sepsis diagnosis is influenced by interactions of factors at micro-, meso-, and macrolevels. Various strategies increased organization-level attention to sepsis, while simultaneously decreasing the provider-level attention necessary for accurate, timely sepsis diagnosis.
- **Meaning:** Accurate, timely sepsis diagnosis depends on leaders cultivating organizational attention that aligns with the conditions that influence providers’ attention to potential sepsis diagnoses.

Sepsis (i.e., life-threatening organ dysfunction caused by infection) remains the costliest inpatient condition and the leading cause of in-hospital mortality in the United States (1–4). Timely antibiotic administration improves sepsis-related mortality (5–7), yet accurate, timely diagnosis remains challenging (8). Nearly half of patients with suspected sepsis are misdiagnosed (9–14), depriving some of lifesaving treatment while exposing others to unnecessary harm (e.g., antimicrobial resistance).

Various organizations have invested substantially in sepsis quality initiatives (e.g., public reporting requirements). The Centers for Medicare and Medicaid Services Severe Sepsis and Septic Shock Management Bundle (SEP-1), a sepsis care composite measure used to determine reimbursement, was established to promote timely sepsis care (15). Hospital initiatives such as electronic health record alerts and enhanced triage protocols are designed to alert providers to potential sepsis diagnoses as soon after presentation as possible (11). Despite these initiatives, diagnostic errors and delays remain major contributors to poor outcomes (9, 10). Variation across EDs highlights the potential influence of complex diagnostic environments on person-, organization-, and population-level sepsis outcomes (16). Few sepsis-related initiatives have consistently improved outcomes, underscoring a critical need to understand why some strategies work, why others do not, and how context impacts strategy implementation (17, 18).

To this end, we developed the Sepsis Diagnostic Excellence (SepsisDx) Theoretical Framework to describe *which* multilevel factors influence accurate, timely sepsis diagnosis. We empirically evaluated and refined the framework using qualitative interviews with sepsis care professionals (e.g., ED physicians, pharmacists, registered nurses) at hospitals throughout the United States. This manuscript reports the refined SepsisDx framework and provides evidence demonstrating *how* these multilevel factors shape real-world diagnostic behavior.

## SEPSIS DIAGNOSTIC EXCELLENCE THEORETICAL FRAMEWORK

SaferDx is a quality and safety framework that conceptualizes diagnostic error through the lens of sociotechnical context (19). SaferDx has been applied to conditions such as appendicitis (20), epidural abscesses (21), and cancer (22). Despite its utility for understanding diagnostic process breakdowns, SaferDx is a general diagnostic safety framework that focuses on diagnosis rather than the individual and organizational dynamics driving diagnosis. Refining SaferDx was thus critical for conceptualizing the unique challenges of sepsis diagnosis.

To conceptualize the unique challenges of sepsis diagnosis, we paired SaferDx with complementary individual- and organization-level implementation frameworks. The Theoretical Domains Framework (TDF) synthesizes 33 psychologic theories into 14 domains representing psychologic influences on individual behavior, representing the behavioral target of sepsis diagnosis among healthcare providers and its psychologic antecedents (e.g., knowledge, skills, attention, motivation) (23). TDF conceptualizes *micro-organizational factors* as individual healthcare providers’ capability and motivation to diagnose sepsis, such as knowledge, skills, attention, and beliefs about the consequences of a sepsis diagnosis (23).

Organization Theory for Implementation Science (OTIS) conceptualizes meso- and macro-organizational influences, including governance, workflows, hospital characteristics, and external regulatory pressures (24).

*Mesoorganizational factors* (from SaferDx and OTIS) comprise the hospital-level environment in which healthcare providers diagnose sepsis, including domains such as hospital governance and operations, and tasks and processes (i.e., organizational initiatives and their antecedents) (24).

*Macroorganizational factors* (from SaferDx and OTIS) represent the broader social, political, economic, and physical context affecting hospitals, such as national COVID-19 pandemic-related staffing shortages, government rules and regulations, and public health emergencies (24).

Together, SaferDx, TDF, and OTIS conceptualize sepsis diagnosis as a clinician behavior shaped by factors at micro-, meso-, and macroorganization levels. A visual representation of the preliminary SepsisDx framework is shown in **Figure 1**. We then refined the SepsisDx framework through the empirical study described below, identifying the subset of domains and constructs relevant to sepsis diagnosis.

**Figure 1.**
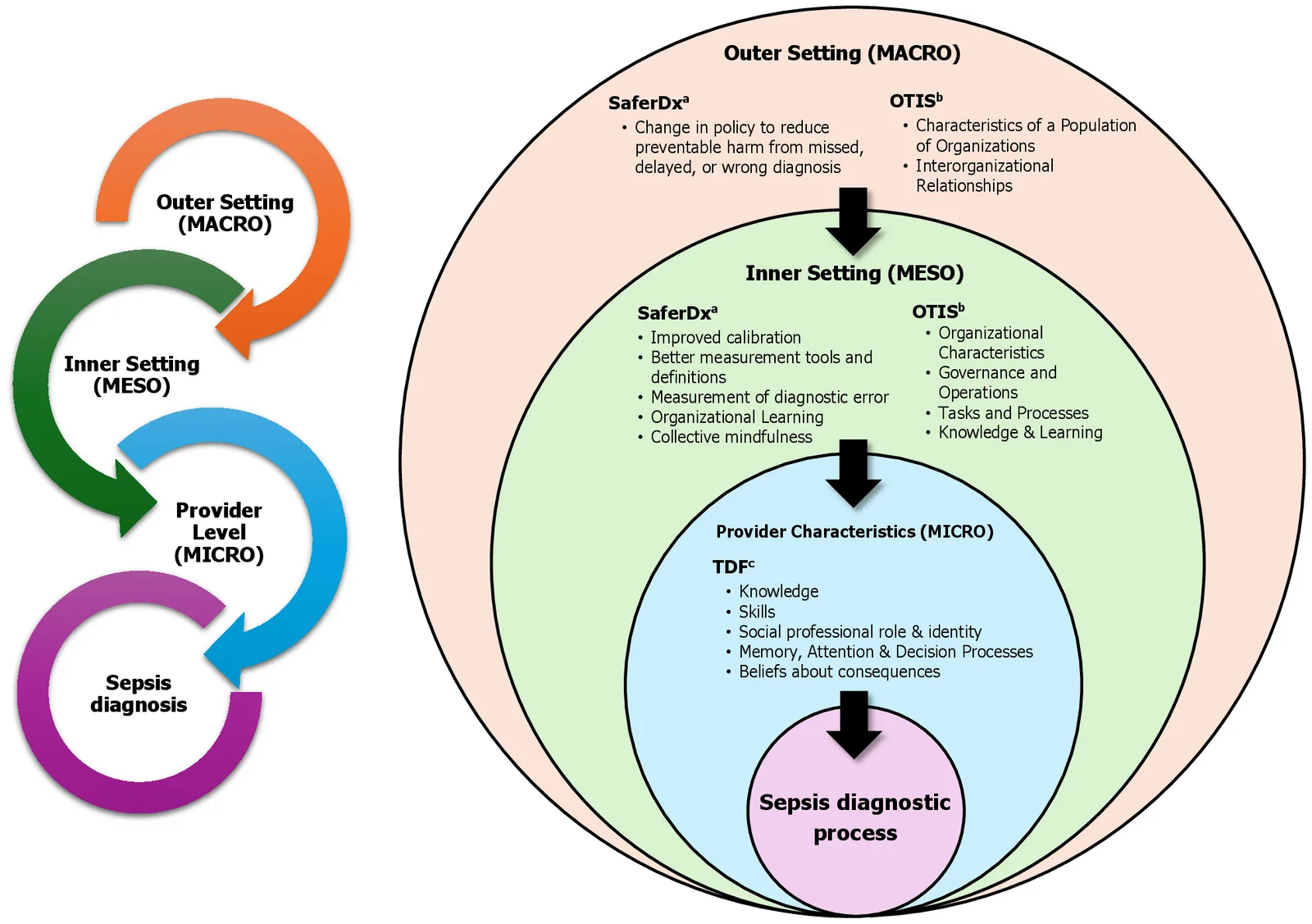
The Sepsis Diagnostic Excellence (Sepsis Dx) theoretical framework. ^a^The SaferDx framework is a quality and safety framework that conceptualizes the sociotechnical context of the sepsis diagnostic process (19). ^b^The Organizational Theory for Implementation Science (OTIS) conceptualizes meso- and macroorganizational influences on the sepsis diagnostic process (21). ^c^The Theoretical Domains Framework (TDF) conceptualizes the psychologic influences on individual provider behavior (20).

## MATERIALS AND METHODS

### Overview

We evaluated and refined the SepsisDx framework using qualitative semi-structured interviews conducted as part of a broader, Agency for Healthcare Research and Quality-funded study to develop guidance on strategies for improving accurate, timely sepsis diagnosis. This study was conducted in accordance with the ethical standards for human research set forth by the University of Michigan Institutional Review Board (IRB) and the Declaration of Helsinki 1975. The University of Michigan IRB approved all study procedures on August 11, 2023 (IRB # HUM00241324).

### Study Setting

We purposively sampled 20 hospitals across four states (AL, GA, MI, NC) and two health systems to maximize representation of location, system structures, and patient populations (**Table 1**). Collectively, these systems account for nearly 500,000 inpatient hospital stays annually across the Midwest and Southern United States. Both health systems had robust clinical quality improvement programs with a special emphasis on improving sepsis outcomes. All hospitals used Epic electronic health record (EHR) clinical platforms. The cross-site variation, common EHR platforms, and large patient volumes were ideal for evaluating factors influencing sepsis diagnosis.

**TABLE 1.** Hospital Characteristics

Site	State	Setting	Beds	Annual Volume	Teaching Status
1	NC	Suburban	457	71,000	Teaching
2	NC	Urban	874	70,000	Academic Medical Center
3	NC	Rural	241	59,000	Nonteaching
4	NC	Rural	67	29,000	Nonteaching
5	NC	Rural	101	40,000	Nonteaching
6	NC	Urban	185	33,000	Nonteaching
7	NC	Urban	307	51,000	Nonteaching
8	NC	Rural	109	31,000	Nonteaching
9	NC	Suburban	182	49,000	Teaching
10	NC	Urban	104	70,000	Nonteaching
11	NC	Urban	885	65,000	Academic Medical Center
12	NC	Suburban	50	29,000	Teaching
13	NC	Suburban	351	53,000	Nonteaching
14	NC	Suburban	94	36,000	Nonteaching
15	NC	Rural	144	29,000	Nonteaching
16	GA	Urban	637	48,000	Nonteaching
17	GA	Rural	140	29,000	Nonteaching
18	GA	Urban	304	60,000	Teaching
19	AL	Rural	60	11,000	Nonteaching
20	MI	Urban	1007	80,000	Academic Medical Center

### Sampling and Recruitment

We sought to interview two to three key informants per hospital based on knowledge of local organizational structures, including top managers (e.g., health system leaders), middle managers (e.g., ED heads; sepsis coordinators [i.e., specialized professional responsible for operationalizing hospital sepsis programs]), and patient-facing providers and staff (e.g., unit nurses and physicians; pharmacists). We asked ED administrative leaders to provide a list of providers and staff who play a key role in sepsis care practice and policy in their ED, ranging from clinical champions to providers and staff without strategic or operational sepsis roles. From the provided information, we identified 54 potential key informants. A study team investigator with established relationships with prospective participants invited them via email to participate in an in-depth videoconference interview. We used purposive and snowball sampling to identify additional key informants. A research assistant followed up with nonresponders via email after one to two weeks, repeating for up to four contacts.

### Data Collection

We developed the interview guide based on SepsisDx constructs (**Appendix 1**, https://links.lww.com/CCX/B690) and refined it through piloting with study team clinicians. S.B., an implementation scientist and experienced qualitative researcher with no previous relationship with study participants, interviewed consenting participants from May to September 2024 via the Microsoft Teams platform. We recruited participants until we reached thematic saturation (25). All interviews were video- and audio-recorded and transcribed verbatim, ranging from 24 to 52 minutes (mean = 35 min).

### Data Analysis

We used a two-pronged analysis approach: 1) S.B. conducted a rapid analysis of interview recordings, with the objective of identifying relationships among the SepsisDx framework’s constructs; and 2) K.R., S.B., and S.S., all trained qualitative researchers, used template analysis (26), applying a priori codes based on SepsisDx constructs and adding emergent codes during analysis in Atlas.ti v24 (Lumivero, Berlin, Germany), a multifunctional qualitative data management and analysis software. K.R., S.B., and S.S. developed a codebook using inductive and deductive approaches based on the frameworks and emerging content from the interviews. They each independently coded transcripts and met to discuss differences and iteratively revise the codebook based on shared understanding. K.R. and S.S. coded the remaining transcripts independently and reviewed each other’s codes. C.W., L.C., S.B., and S.S. identified themes (i.e., declarative statements describing causal relationships between a given construct and the outcome) within codes. We collaboratively identified themes for one construct and then independently identified themes for the remaining constructs, regularly meeting to resolve discrepancies. We refined preliminary findings through study team discussion and a member-checking exercise in which we solicited feedback on theme validity from our seven-person study advisory board and all 41 interview participants. Minor revisions were made to themes to better reflect participant experience.

## RESULTS

Interviews were conducted with 41 sepsis care professionals from 13 hospitals (**Table 2**). Thirteen targeted key informants representing seven hospitals did not respond to repeated invitations.

**TABLE 2.** Participant Characteristics

Characteristics	*N* = 41	%
Clinical role		
Physician	6	14.6
Physician director or administrator	12	29.3
Quality physician	3	7.3
Nurse	2	4.9
Nurse manager or administrator	10	24.4
Sepsis coordinator or nurse educator	6	14.6
Pharmacist	2	4.9
Location		
System or market-level	7	17.1
Multiple hospitals	5	12.2
Individual hospital	29	70.7
Department or specialty		
Emergency	28	68.3
Quality or patient safety	4	9.8
Administration	2	4.9
Pharmacy	2	4.9
Pulmonary or critical care	2	4.9
Other (internal, infectious disease, inpatient)	3	7.3

### Overview

Interview data validated the central premise of SepsisDx: sepsis diagnosis, conceptualized as an individual clinician behavior, is determined by the interaction of factors at micro-, meso-, and macrolevels. Individual-level knowledge and attention are necessary but insufficient for accurate, timely sepsis diagnosis. Individual factors were strongly influenced by conditions within clinical teams, EDs, hospitals, and health systems. Below, we present interview data as primary evidence for the framework (**Appendix 2**, https://links.lww.com/CCX/B690).

### Microorganizational Factors

#### Knowledge of Sepsis Signs and Symptoms

Participants linked diagnostic performance to providers’ knowledge of sepsis signs and symptoms and experience engaging with patients with suspected sepsis:

“It comes down to the experience …to be able to recognize the not-so-obvious signs that someone may be septic.” (ED Nurse, Hospital 13)

Leaders often sought to improve knowledge through training and education that was relational (i.e., knowledge-sharing among individuals and groups), didactic (i.e., formal training), and experiential (i.e., engaging with patients with suspected sepsis), without pursuing strategies to address other influences:

“Education [is] probably 85 percent of what we’re doing…whatever modality we can.” (ED Director, Hospital 14)

“When there are changes in sepsis care, we have standard meetings…a department meeting…email education... Oftentimes, there has to be a little bit of at-elbow support and training for computer stuff.” (Physician Administrator, Hospital 16)

#### Attention to Sepsis as a Potential Diagnosis

Attention emerged as the most salient individual-level factor influencing sepsis diagnosis (i.e., focus on one out of many possible tasks or trains of thought) (27). Ambiguous clinical presentation reduced attention and impeded diagnosis:

“If people walked in with a sign on their forehead saying, ‘I have sepsis,’ it’d be easy…but you’ll get patients who come in who just don’t feel good…then as labs come back, you realize…this actually is a sepsis patient.” (ED Director, Hospital 14)

Participants reported that COVID-19 further diverted attention as COVID-19 care protocols acted as a competing priority and diminished providers’ ability to comply with sepsis-related recommendations and requirements, straining resources (e.g., staff, equipment) and demoting sepsis as a clinical priority.

Attention was also influenced by collective mindfulness—that is, everyone and every system working in concert toward quality sepsis care. Collective mindfulness was facilitated by consistent messaging regarding sepsis as a priority, multidisciplinary collaboration, and communication support tools (e.g., EHR documentation, huddles). Tools’ effectiveness was diminished by outcomes that were insufficiently tied to sepsis-related processes and competing clinical and administrative demands. Process measures (e.g., order set use) were not directly tied to patient outcomes (e.g., mortality). For example, “sepsis swarms” (28, 29) increased awareness of sepsis as a potential diagnosis by facilitating more rapid patient evaluation by multiple care team members but required alignment with health system metrics:

“We do [sepsis swarms] much earlier…and more often …so that when we do call a Code Sepsis, people have done it recently… We tried to preserve the usefulness of the ‘swarm’ while still trying to meet the <health system> metrics, but also making it match more with our clinical needs.” (Quality Physician, Hospital 16)

### Mesoorganizational Factors

Primary hospital-level factors influencing sepsis diagnostic behavior included hospital and health system characteristics and strategies intended to increase attention to sepsis. We grouped strategies (**Table 3**) into the following domains:

**TABLE 3.** Hospitals’ Sepsis Diagnosis-Related Strategies

Strategies
Workflow modifications ○ Nurse-driven protocols (i.e., is it within a nurse’s scope of responsibility in the hospital to initiate a care process for suspected sepsis) ○ Code sepsis ○ Antibiotics stored in the ED (e.g., Omnicell) ○ Sepsis swarm ○ Sepsis alerts ○ Front desk triage protocols ○ Infected persons pathway
• Communication ○ Handoff tool form ○ Sepsis screening questions administered to all patients at first contact ○ Sepsis huddles ○ Sepsis awareness-raising campaigns (e.g., buttons, giveaways, prizes) ○ System-level audit and feedback (i.e., process of delivering sepsis quality data to healthcare providers) ○ Individual-level feedback from chart reviews/fallouts ○ Chart reviews ○ Meetings (sometimes with incentives for participation) ○ E-mail ○ Dashboard scorecards ○ Chart documentation ○ Bedside, real-time teaching ○ Clinician co-location ○ Clinician-to-clinician phone calls (e.g., ED to pharmacy) ○ Secure chat ○ Sepsis-related trainings and education (online and in-person and Flip Classroom; for new staff and refreshers for established staff) ○ Public kudos (e.g., “Sepsis Stars”) ○ Posters with sepsis diagnostic criteria ○ Badge buddies with sepsis diagnostic criteria ○ Mouse pads with sepsis diagnostic criteria
• Leadership initiatives ○ Sepsis committees ○ Incentives (pay bonus, gift card, public recognition) for sepsis-related performance ○ Antimicrobial stewardship efforts are part of decision-making around treatment for suspected sepsis
• Sepsis-related personnel ○ Sepsis champions ○ Sepsis coordinators ○ Pharmacist present in the ED or on call ○ Engaging multidisciplinary leaders representing hospitals across the health system in sepsis-related strategy development and implementation

ED = emergency department.

#### Technology & Workflow Modifications

Strategies, often EHR-based, such as nurse-initiated order sets and sepsis triage screening, were commonly used. Providers, however, described substantial limitations of electronic sepsis diagnostic support, with many expressing a desire for automated detection tools but noting that the technology was not yet sufficiently sophisticated:

“They call this workflow ‘Sepsis 2.0’…triage nurses…positive screen…order lactate, blood cultures…” (Sepsis Coordinator, Hospital 20)

“We put a lot of work into order sets…and then nobody uses them…It just includes too much.” (ED Director, Hospital 19)

“[O]ur tools for detection are either too sensitive or too nonspecific.” (ED Director, Hospital 11)

#### Communication Strategies

Hospitals used tools such as provider audit and feedback to draw attention to “fallouts”—that is, patients who were ultimately diagnosed with sepsis but not by the ED provider or whose care was not documented in accordance with SEP-1 criteria. Providers noted that significant lags between performance data entry and review (6–18 mo) diminished performance report credibility and affected their ability to recall and address concerns.

“It’s frustrating…Most of the misses we have are [because] we are a busy [ED]…even if they gave [antibiotics], they didn’t scan it until five minutes after…now we missed our measure.” (Sepsis Coordinator, Hospital 6)

#### Leadership Initiatives

Participants suggested that multidisciplinary leadership initiatives, such as sepsis committees, enhanced collective mindfulness:

“We just had our first inaugural sepsis committee meeting last month. That is an opportunity to talk about all of the metrics and how well we’re continuing to care for sepsis patients.” (Sepsis Coordinator, Hospital 16)

“We…have forums to review new data, literature, practices, and discuss and collaborate with the emergency department of, ‘do we [want to] make this change? Do we [want to] make changes to our order sets based on it?’” (Critical Care Physician, Hospital 7)

#### Sepsis-Related Personnel

Participants reported that sepsis coordinators’ real-time feedback and ED-embedded pharmacists improved attention by supporting diagnostic and treatment decisions:

“[A] sepsis coordinator would provide individualized feedback on sepsis patients…to providers and the team…so we would get close to real-time feedback on individual cases.” (Physician Administrator, Hospital 2)

“[Pharmacists] help us with the sedation drips… provide education and keep us updated on drug shortages and any changes. Also, if the physicians are not sure about a medication, they can just go to them and talk with them.” (ED Nurse Manager, Hospital 16)

### Macroorganizational Factors

SEP-1 criteria shaped sepsis-related best practices, pressuring health systems to develop strategies such as best practice advisories, order sets, and sepsis huddles to improve compliance. SEP-1 increased organizational awareness but competed for limited provider and staff attention, with multiple participants reporting that SEP-1 often conflicted with clinical judgement.

“[W]e’re currently…trying to navigate…all of the subsequent administrative pressures to perform well on SEP-1 from a hospital-administrative perspective because it contributes to so much publicly reported information and financial reimbursement.” (Physician Administrator, Hospital 2)

“We’re asking [nurses] to do so many other things…they feel taken away from really good bedside nursing.” (ED Director, Hospital 14)

“Physicians say, ‘Well, [SEP-1 is] not evidence-based, and we don’t necessarily agree with it.’” (Quality Manager, Hospital 2)

### Moderators: Organizational Characteristics Affecting Strategy Effectiveness

Strategies’ influence on attention to sepsis was moderated by the extent to which health systems prioritized strategies, how engaged hospital and ED leaders were in implementing sepsis-related recommendations and requirements, and whether necessary diagnostic resources were available.

#### Hospital Size and Type

System-level leaders suggested that larger academic hospitals and health system hubs more often used strategies and dedicated resources to managing patients with suspected sepsis, but faced challenges due to clinical learners who lacked the time to become proficient in hospital sepsis protocols and providers who prioritized clinical autonomy over compliance:

“It’s the curse of academia because they understand the literature behind it. Then I might get my community setting where if this is what the order set says, let’s just go ahead and get it done.” (Physician Administrator, Hospital 11)

Rural hospitals were described as having lower patient volumes, limiting provider and staff opportunities to become familiar with sepsis signs and symptoms and procedures for implementing sepsis protocols. Rural hospitals also tended to be less integrated into health systems, diminishing the relevance of system-level sepsis-related strategies.

#### Patient Populations

Rural patients often arrived at rural hospitals with more critical health status, prompting providers to initiate more rapid and aggressive treatment. Rural patients were often uninsured, at times compromising early intervention for suspected sepsis.

“Those that don’t [have insurance] are less reluctant because of the financial burden, so they may present to the ED a lot sicker than others would.” (ED Nurse Manager, Hospital 15)

#### Staffing Shortages

National staffing shortages, exacerbated by the COVID-19 pandemic, reduced diagnostic attention by creating knowledge gaps with increasing turnover rates as experienced staff resigned:

“If we’re short staffed, it makes it harder…to get a group in that room to start taking care of the patient.” (ED Nurse Manager, Hospital 13)”

#### Resources

Participants reported that sepsis diagnosis improved when health system strategy aligned with hospital-level resources and activities and noted variation in hospital performance that reflected access to resources.

“Resources—any of the community hospitals is [going to] come down to resources. When I’m at <academic hospital>, I have a pharmacist that’s on my shift with me. That’s unheard of at a community hospital...They actually can look and say what antibiotics they need for sepsis. At a community hospital, I’m trying to figure this stuff out.” (Physician Administrator, Hospital 2)

**Figure 2** displays the subset of constructs included in the preliminary SepsisDx framework (Fig. 1) that interview participants identified as relevant to sepsis diagnosis.

**Figure 2.**
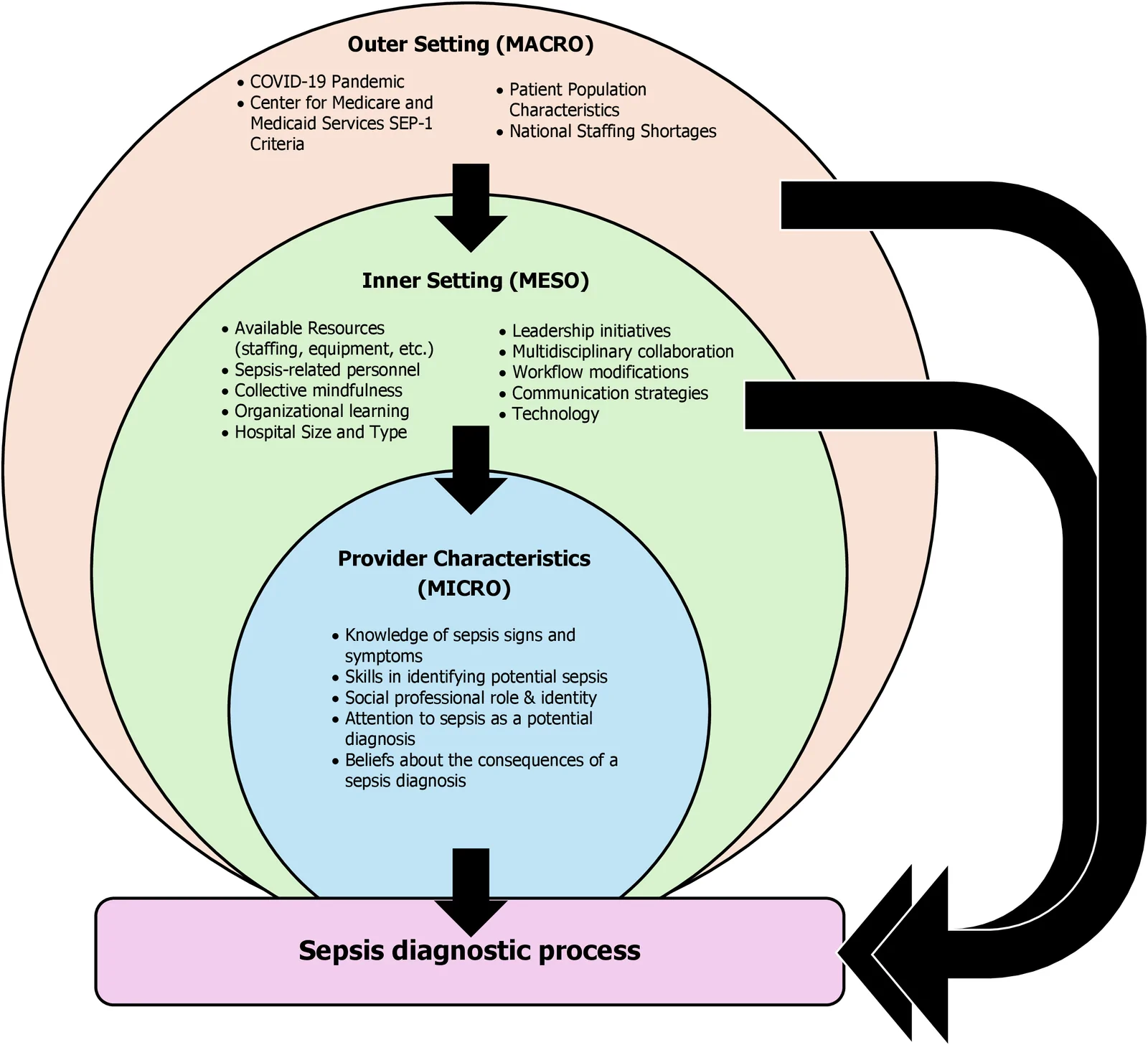
The refined Sepsis Diagnostic Excellence framework.

## DISCUSSION

In this multi-site qualitative study, we integrated three extant frameworks, SaferDx, TDF, and OTIS, to develop a comprehensive theoretical framework, SepsisDx, to describe how multilevel factors influence accurate, timely sepsis diagnosis. We then tested SepsisDx across interviews with 41 sepsis care professionals. The resulting framework clarifies *which* factors affect sepsis diagnosis and *how* these factors operate through specific mechanisms, representing an advancement beyond existing frameworks (19, 30).

Our findings demonstrate that the core behavioral mechanism linking micro-, meso-, and macrolevel influences to diagnostic performance was attention to sepsis as a potential diagnosis (**Fig. 3**). Sepsis may not occupy clinicians’ attentional foreground until confirmatory data emerge, potentially delaying critical interventions. Participants repeatedly emphasized the challenge of identifying sepsis amid ambiguous presentations, high-acuity environments, and competing cognitive demands, consistent with prior studies (31).

**Figure 3.**
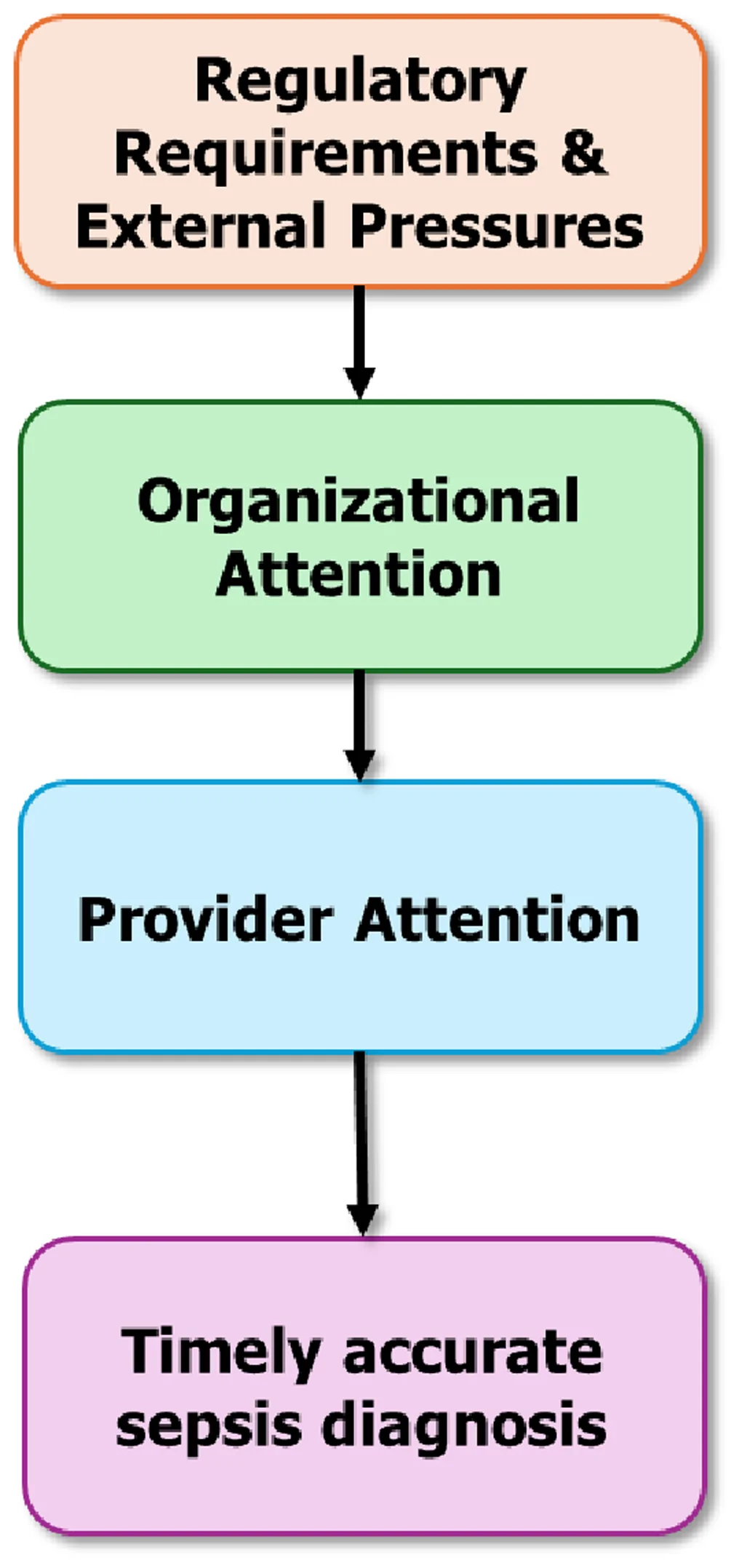
Central mechanisms of influence on Sepsis diagnosis.

Clinicians’ attention to sepsis as a potential diagnosis was often distracted by *organizational attention*—that is, collective-level focus comprised of organizational stimuli constructed by leaders and upheld by providers and staff (32–34). Sepsis leaders (e.g., chief medical and nursing officers) translated macro-level influences (e.g., SEP-1) into organizational stimuli (e.g., sepsis care protocols, audit and feedback). In turn, organizational stimuli were overseen by mid-level managers (e.g., medical directors, physician leads, nurse managers), whose efforts were also influenced by meso-level factors (e.g., available staff), and directed at clinicians, whose behavior was also influenced by micro-level factors (i.e., beliefs about the consequences of SEP-1 criteria adherence). Organizational attention focused on addressing macrolevel influences often conflicted with meso- and microlevel influences, representing a displacement of goals with heuristics and metrics (35) commonly observed in organizations. Displacement occurs when bureaucracies codify goals in policies and procedures that lack the specificity required for operationalization, leaving them subject to distortion in practice (36). Conditions of high ambiguity, including sepsis diagnosis, are particularly vulnerable to goal displacement.

Leaders construct attention economies that are encoded in and modified by hospital and ED social, technical, and governance systems (33). Critically, the organizational attention that social, technical, and governance systems yield has properties that are *independent of* individual provider-level attention to potential sepsis diagnoses. Thus, even when organizational attention to potential sepsis is high, sepsis may go undiagnosed because individual provider attention is diffuse (31). In fact, high organizational attention often decreased individual attention. For example, educational initiatives comprised a significant portion of sepsis organizational systems yet were viewed as repetitive and detached from the contextual pressures that most directly shape real-time diagnostic behavior. Similarly, SEP-1 increased organizational attention to sepsis, yet documentation requirements and compliance-oriented strategies decreased providers’ attention to potential sepsis diagnoses, consistent with Winslow (37). We extend Winslow’s findings by characterizing the bureaucracy that drives metrification, pointing toward strategies for improvement. Indeed, initiatives such as ED sepsis coordinators, code sepsis, and sepsis huddles garnered organizational attention to sepsis consistent with provider-level attention. This finding aligns with evidence that proactive, communication-focused strategies improve sepsis care (31).

SepsisDx offers a more nuanced alternative to existing guidance on improving sepsis diagnosis (38). SepsisDx may help leaders cultivate social, technical, and governance systems that reflect clinicians’ lived experience attending to sepsis as a potential diagnosis. For example, chief medical officers could modify system-level metrics to account for local context (e.g., volume, staffing) or tailor organization- and ED-level systems to improve individuals’ receptivity to health system-level strategies (e.g., real-time feedback on health system metrics). Clinicians’ attentional bandwidth increased when systems for attending to macro-level influences (e.g., SEP-1) were centralized and systems for attending to meso- and microlevel influences were decentralized. Indeed, one physician lead wished for templates to communicate health system-level sepsis initiatives instead of having to “reinvent the wheel at each hospital”; others were frustrated by system-developed order sets that were incongruous with hospital-specific workflows. These findings align with emerging evidence suggesting that, to produce desired outcomes, interventions and implementation strategies must be tailored to unique contexts (39).

Finally, understanding organizational factors, their interactions, and influences on person-, organization-, and population-level sepsis outcomes is an important yet understudied area of sepsis care delivery research. The field has thus far lacked conceptual grounding to guide researchers. The organizational factors described here likely influence whether and how clinical discoveries improve sepsis diagnosis, treatment, and outcomes. Thus, SepsisDx provides important insight into the organizational factors relevant to sepsis diagnosis that should be included in research evaluating interventions to improve outcomes. In particular, future studies should test four SepsisDx propositions: 1) As macrolevel attention (in this case, attention to reimbursement) migrates to provider level, individual attention migrates from diagnostic accuracy toward documentation compliance. 2) Sepsis diagnosis improves with increased alignment of attention at macro-, meso-, and microlevels. 3) Alignment of macro-, meso-, and microlevel attention improves with increased collective mindfulness. 4) Centralized responses to macrolevel pressures (e.g., system-level metric management, templated communications) and decentralized meso/micro-attending systems (e.g., locally adapted order sets, local feedback) improve sepsis diagnosis. Several limitations warrant consideration. We focused on community-onset sepsis with the ED as the diagnosis setting, and our framework may not be generalizable to the diagnosis of hospital-onset sepsis. In addition, findings may not be transferable beyond study sites, which all used Epic and were resource-rich relative to the constraints of many US EDs; however, included hospitals represented a broad geographic swath of the United States and several institution types (e.g., academic, community). Further, despite rigorous recruitment efforts, we were unable to recruit participants from seven of the 20 potential hospitals and may not have captured all perspectives at participating sites, including those who were unable or unwilling to participate. Notably, however, sites from which we were unable to recruit participants were similar to included sites in terms of size, location, and type, and included sites represented all study states and markets. It should also be noted that we integrated three evolving frameworks that may lack key determinants of accurate, timely sepsis diagnosis. Indeed, we added the Attention-based View of the Firm to OTIS post hoc because OTIS lacked organizational attention as a construct. SepsisDx should be subjected to similar revision through empirical application.

Overall, this work advances sepsis diagnostic research by clarifying pathways through which multilevel determinants shape real-time decision-making, facilitating intervention tailoring to highly varied sepsis care contexts. By grounding theory in lived experience across diverse ED settings, the refined SepsisDx framework offers a robust foundation for designing, implementing, and evaluating strategies that meaningfully improve the accuracy and timeliness of sepsis diagnosis. Future research should validate SepsisDx in other settings.

## ACKNOWLEDGMENTS

We gratefully acknowledge the contributions of our study advisory board, the 41 sepsis care professionals who participated in this research, and feedback from Larry Hearld and colleagues at the 2026 Organization Theory in Health Care conference.

## Supplementary Material


