# Supplementary material for: Sepsis Diagnostic Excellence: Development and Validation of a Theoretical Framework for Conceptualizing Accurate, Timely Sepsis Diagnosis
Source: Crit Care Explor. 2026 Sep 25;8(10):e1490. doi: 10.1097/CCE.0000000000001490 (PMC13619180; doi:10.1097/CCE.0000000000001490)
Supplement: Supplementary file 1 [file cc9-8-e1490-s001.pdf]

Supplemental Materials

Table of Contents

Appendix 1. SepsisDx Framework Qualitative Interview Guide..2

Appendix 2. Themes organized by SepsisDx Framework domains and constructs..............4

Appendix 1. SepsisDx Framework Qualitative Interview Guide

[Interviewer review participant's open-ended survey questions]

Is this still a good time for you? Are you in a place where you can be free from distractions and feel free to give candid responses?

Thank you for agreeing to speak with me today. The purpose of this interview is to help us understand your hospital's approach to improving sepsis diagnosis and treatment and the things that make that approach work or prevent that approach from working. Fundamentally, we don't want to miss sepsis, but we also don't want to treat things that turn out not to be sepsis (e.g., due to antimicrobial stewardship concerns). We are interested in learning about things that are happening in your hospital that help to strike this balance and things that represent a distraction from reaching this balance.

We will be using the information you share with us today to help construct site-based profiles of approaches to improving sepsis diagnosis and treatment. While we will be keeping your location tied to your interview, we will remove any personal identifiers so that you will remain confidential in what you share with us. With the information we gain from these profiles, we hope to develop more effective interventions to support high-quality care for patients. Our discussion should last about 30-45 minutes. You may choose to stop the interview at any time, and there is no penalty to you for not completing the interview.

Taking part in this research is not a part of your job duties, and refusing will not affect your job. You will not be offered or receive any special job-related consideration if you take part in this research. Before we begin, we would like to ask your permission to audio record our discussion. The recording will be used for training and transcription purposes. The audio recordings will be deleted once the project is complete. If you have any questions, I can provide contact information for the **PI [sbirken@wakehealth.edu].**

Do you have any questions before we begin?

Also, I'd like to record this interview for the purposes of analysis. The only people who will have access to the recording or subsequent transcript are study personnel who are trained in maintaining confidentiality in human subjects research. Are you okay with being recorded?

If yes, hit record now.

Could we start with you telling me who you are and what your role is at your hospital?

1. Thinking back to 2021-23, what would you say your hospital's approach was to striking that balance between diagnosing and treating all cases of sepsis while not inadvertently treating those without sepsis?
   a. How stringently does your hospital approach sepsis? (e.g., treat all suspected sepsis no matter what; be really cautious about treating things that might not be sepsis) On a scale of 10 is treat all suspected sepsis no matter how likely and 1 is being really cautious about not

treating sepsis when it is not present, where would you say your hospital falls? Why would you give that rating?

2. What kind of things do you think predisposed your hospital to that approach? (e.g., resources, rules/regulations, time, staffing, professional culture [e.g., anti-‘cookbook’ medicine])
3. What kinds of tools did your hospitals use to support that approach? (e.g., training, order sets, BPAs/pop-up HER alerts, probability models/clinical risk modeling/sepsis sniffers/HER screening etc.)
   a. What would a practicing provider or nurse or pharmacist’s experience be of your hospital’s approach?
4. How was your hospital’s approach developed? (e.g., based on CMS regulations, professional society recommendations, what peer institutions are doing; antimicrobial stewardship campaigns)
   a. Who participated in developing it? Who did not participate? Do antimicrobial stewardship campaigns and sepsis quality work together on it?
   b. What tasks were involved in developing it?
   c. What went well about the development process? What didn’t go so well?
5. How was your hospital's approach communicated to providers, staff, patients, and caregivers? E.g., meetings, emails, grand rounds, job descriptions, required training
   a. In what ways does the approach to communication work well? Not so well?
   b. Whom does the communication reach? Whom does it not reach?
6. What kind of efforts, if any, are there in your hospital to enforce or encourage providers and staff to follow its approach? (e.g., incentives, performance metrics)
7. Tell me about your hospital approaches to incorporating new knowledge about sepsis (e.g., from its own performance or other hospitals’ performance, evidence or new guidelines) into its approach?
   a. Tell me about any monitoring that your hospital does around sepsis processes and outcomes.
   b. What kind of feedback, if any, is shared with providers, staff, patients, other stakeholders? How is it communicated, how often? Is it tied to performance reviews or incentives or any other consequences?
   c. What works well about that? What doesn't work well about that?

CLOSING

Those are all of the questions that I had planned for today. Is there anything else that you want to tell me that would help me to understand how you aim to make accurate sepsis diagnoses? Thank you for your time!

Appendix 2. Themes organized by SepsisDx Framework domains and constructs

Construct	Theme	Example quotes
Sepsis Diagnostic Process (SaferDx)		
Patient-provider encounter	The goal of the patient-provider encounter was to decrease time to recognition of sepsis. It was characterized by the provider orienting to the patient and assessing risk for sepsis based on symptom presentation and pre-existing conditions (e.g., heart failure). Characteristics of hospitals' patient populations (e.g., prevalence of chronic conditions) sometimes complicated sepsis risk assessment. Tools intended to decrease time to treat sepsis (e.g., EHR alerts, "sepsis sniffers," nurse-driven protocols, SEP-1) were sometimes counterproductive (e.g., clicking past alerts; documentation for SEP-1 compliance).	"Yeah. I'd say challenge is diagnostic uncertainty. If people walked in with a sign on their forehead saying "I have sepsis," it'd be easy to just throw all the sepsis-specific orders in and order their antibiotics and move along, but you'll get patients who come in who just don't feel good and they don't actually have a fever, and then as labs come back, you realize, "Oh, they actually have a pneumonia, and their white blood cell count's terrible and they're dehydrated, so this actually is a sepsis patient even though it didn't look like it initially...you realize later in the visit, "Well, this one's gonna be a fallout because we didn't do the stuff that we didn't know we were gonna need because we didn't need it yet, and now they do." (ED Director, Hospital 14)
Initial diagnostic assessment	Sepsis is challenging to diagnose, contributing to inaccurate diagnosis and challenges adhering to SEP-1. In many cases, nurses were the first to screen for sepsis; a nurse-driven sepsis protocol improved early sepsis detection.	"There's a lot of confusion around sepsis, when to call it sepsis, when not to. I've seen a lot of people put sepsis on the chart when there's no infection, and it just don't make a lot of sense to me. I'm just trying to—we're getting better at it. If I understand it, I can teach it, but I don't really fully understand it myself. I don't know." (Physician Administrator, Hospital 18)<br><br>"We're working on a more of a nurse-driven, "I've got an infection plus this. We're going to go ahead and call it."" (ED Nurse, Hospital 18)
Diagnostic test performance/ interpretation	Barriers to accurate sepsis diagnosis included:<br>• Ambiguous clinical presentation<br>• Significant hospital resources (e.g., human, financial, time) associated with sepsis-related strategies that made providers reluctant to diagnose sepsis<br>• Beliefs that recommendations and requirements do not align with best practices for patient care	"Stroke and myocardial infarction are discrete entities with gold standard tests and pathologic correlates. That does not exist for sepsis, because again, like a ischemic stroke, different parts of the brain, different causes, but in the end, it is something is blocking a blood vessel. You have death of brain tissue. Myocardial infarction, something is blocking a blood vessel. You have death of cardiac tissue. You can measure that. You can see it, whereas sepsis is a thousand different things." (Nurse Educator, Hospital 11)

	Factors thought to facilitate accurate sepsis diagnosis included:<br>• Nurse-driven protocols<br>• "Code sepsis" protocols<br>• System or hospital-level (not individual provider-level) audits to understand barriers to compliance with recommendations and requirements<br>• Initiating workup and diagnosis prior to arriving at the ED (e.g., by emergency medical services, first responders)<br>• "Sepsis sniffers"<br>• Accurate screening (e.g., clean blood cultures)<br>• Ensuring receipt of screening results (e.g., blood cultures) prior to administering antibiotics	
Subspecialty consultation/ referral issues	Patients were vulnerable to lapses in care when subspecialists lacked the time to review individual patient cases and during transitions between specialties. Lapses in patient care related to transitions between specialties were mitigated by clear communication and access to resources (e.g., ICU).	"[W]e have what we call HOT forms. They're hand-off tools, and it's a form that has all of the sepsis criteria for SEP-1, so did you get your lactic acid, what is your time zero, did you draw your blood cultures before your antibiotics, the time the antibiotics were given—because we do have a goal of administration within 60 minutes of identification—and then the amount of fluids that were given. That is the tool that is handed to the inpatient nurse when they go upstairs so they know what they need to do next or what was missed or how many more—how much fluids they need after they transition to inpatient. They do have the HOT form. They have adult standing orders for the ED, and then they have order sets." (Quality Manager, Hospital 2)
Follow-up and tracking of diagnostic information	Hospitals used various tools (e.g., dashboard scorecard; HOT form compliance reports; sepsis-focused personnel assignments) for <u>tracking</u> diagnostic information. Processes for <u>follow-up</u> based on tracking included:<br>• Regular meetings with individuals or teams to discuss concerns<br>• Emails to provide feedback to providers<br>• Audit and feedback<br>• Recognition of successes (e.g., public kudos; awards)	"We would love to look at every single fallout and then give a detailed email about what, but it's just so much work. You can't do it." (ED Physician, Hospital 2)<br><br>"[W]e have a nurse leader and then three additional nurses, each one dedicated to a particular environment, adult hospitals, children's and women's and the emergency department, who spend all their time—we do 100 percent review of cases. We send out kudos." (Quality Director, Hospital 20)

	• Bedside/real-time education and feedback<br><br>Barriers to the efficacy of follow-up and tracking tools and processes included lack of sepsis-specific personnel (e.g., sepsis coordinator) and non-overlapping work schedules (e.g., night shifts).	
Individual-level influences (Micro; TDF)		
Knowledge	New nurse graduates were not familiar with diagnostic criteria.<br><br>EDs over-relied on strategies that targeted knowledge and do little (or nothing) to address other individual-level influences and influences at collective levels (e.g., health system influences).	"Generally, new grads do go through a specific program where they get trained on the typical things we see in the emergency department and how to handle it. I think at times, what it comes down to is the experience with patients, to be able to recognize the not so obvious signs that someone may be septic. I think a lotta times, that's where the holes are in the care that we give. It's just the nurses' experience and ability to identify it." (ED Nurse, Hospital 13)<br><br>"I think it is an opportunity for everybody to understand that maybe the patient was septic. They're really using the generic terms like urosepsis, pneumonia, infection from somewhere else, as the principal diagnosis. I think charting was a big problem…I think it all gets back to the knowledge base and the training piece there." (Hospitalist, Hospital 15)
Motivation and goals	Tools intended to decrease time to treat sepsis (e.g., electronic health record alerts, "sepsis sniffers," nurse-driven protocols, SEP-1) were sometimes counterproductive (e.g., clicking past alerts; documentation for SEP-1 compliance).	"For something like sepsis, these are some of our sickest patients, and so if you can clearly state the value proposition to all parties of like, 'This is the value that the change will bring to you as a worker and then to our patients,' they're not hesitant, by any means." (13-2)
Social influences	In addition to hospitals' access to resources (e.g., ICU; pharmacists), the efficacy of sepsis-related strategies was influenced by:<br>• Coordination among system, regional, hospital, and ED priorities<br>• Collaboration among sepsis-related leaders, providers, and staff	"If you need the support of the providers, I think education is a big piece. I think before we initiate or we do anything different here, there should be a lot of education. There should be a lot of buy-in of all the stakeholders before we implement anything." (Hospitalist, Hospital 15)
Professional role and identity	Compliance with sepsis-related recommendations and requirements was variable due to provider beliefs that metrics did not align with best practices for patient care.	"[T]here's still some hesitancy about following exactly the SEP-1 bundle criteria, and I've heard physicians say, 'Well, it's not evidence-based, and we don't necessarily agree with it, but we have

		to implement it and we have to support it because of CMS.' There's some back-and-forth about doing what's required by CMS and doing what's evidence-based, so there's—I've just heard comments back and forth from a handful of providers about whether they support or believe in the SEP-1 bundle or not." (Quality Manager, Hospital 2)
Habits and routines	Hospitals sought to bridge the gap between sepsis-related strategies (e.g., SEP-1) and what occurred in clinical practice with strategies such as order sets and training. The efficacy of these strategies was limited by (1) the dynamism of both sepsis-related strategies and of clinical practice and (2) the burden of documenting clinically permissible deviations from sepsis-related strategies in practice.	"We used to review that in the Performance Improvement Committee, PIC. If anybody overdid it, anybody underdid it, or if a particular patient, if a particular provider is using too many antibiotics, it's just the habit of doing things. It's not necessarily what's the correct antibiotic. It's just the habit of doing things." (Hospitalist, Hospital 15)
Competing task demands	Sepsis-related personnel's behavior (i.e., engaging in sepsis care; applying strategies and workflow and communication tools) was constrained by demands associated with other clinical and administrative tasks.	"I think we're asking them to do so many other things than bedside nursing …for example, we need you to make sure that you're checking on the patient every 15 minutes and documenting, that all your stroke scales are in, that you're getting antibiotics started within an hour…they take them away from what they were wanting to do, which is really good bedside nursing where you spend the time talking with families, taking care of patients…(ED Director, Hospital 14)
Beliefs about consequences	Barriers to accurate sepsis diagnosis included beliefs that recommendations and requirements did not align with best practices for patient care.	"You're increasing the risk of antibiotic-associated complications in the patient. You might hospitalize them unnecessarily…so over-testing and over-treating is a harm. If you've got a patient with a history of heart failure and of pneumonia, and you decide that they're septic and you give them 30 CC per kilogram of bodyweight of IV fluid, you're going to cause substantial risk for harm because their heart can't handle that much fluid." (ED Nurse Manager, Hospital 15)<br><br>"Even with infinite resources, infinite nurses, infinite beds, treating every minor infection like sepsis is not good for the patient." (ED Nurse Manager, Hospital 15)

		"Now, it could be fairly dramatically bad if that patient then develops C difficile colitis...That is possible. That is a really bad outcome of inappropriate antibiotic use." (Pharmacist, Hospital 11)<br><br>"I think there's more fear in working someone up for sepsis than there's more confidence for working up patients with sepsis because of the fear of fluid overload, the fear of injuring kidneys and the fear of providing antibiotics when everyone seems to want antibiotics…" (Pharmacist, Hospital 11)
Nature of the behavior	Reasons for non-compliance with sepsis metrics (e.g., SEP-1) included ambiguous clinical presentation.	"There's a lot of confusion around sepsis, when to call it sepsis, when not to. I've seen a lot of people put sepsis on the chart when there's no infection, and it just don't make a lot of sense to me. I'm just tryin' to—we're getting better at it. If I understand it, I can teach it, but I don't really fully understand it myself. I don't know." (Physician Administrator, Hospital 18)
Inner Setting (Meso; OTIS)		
Internal organizational features	External pressures drove hospitals' sepsis-related strategies. Sepsis-related strategies were influenced by:<br>• System and regional leaders seeking to streamline care across hospitals<br>• Hospital leaders seeking to optimize performance with respect to metrics<br>• ED leaders seeking to elevate opportunities for improvement to leaders<br><br>The efficacy of sepsis-related strategies was influenced by:<br>• Coordination among system, regional, hospital, and ED priorities<br>• Collaboration among sepsis-related leaders, providers, and staff<br>• Hospitals' access to resources (e.g., ICU; pharmacists)	"Resources—any of the community hospitals is gonna come down to resources. When I'm at <academic hospital>, I have a pharmacist that's on my shift with me. That's unheard of at a community hospital…" (Medical Director, Hospital 15)<br><br>"I knew that there was some newer sepsis initiatives here at <academic hospital>, and when I talked to them at <community hospital>, it sounds like they're creating their own sepsis committee. People on the sepsis committee—I think they are trying to do an independent review as opposed to just, 'Well, we'll just see what the institution says, and we'll do that.' Yes, many things from a leadership standpoint. Initiatives are local." (ICU Physician, Hospital 14)
Collective mindfulness	Collective mindfulness (i.e., everyone and every system working in concert toward quality sepsis care) was facilitated by:	"I think just streamlined the process from when the patient first step in the door—having that collaborative team approach from registration to pre-hospital provider for nursing staff...Just having a more open communication so it's—the care team is not so much

	• Consistency in the content and delivery of messaging regarding sepsis as a priority<br>• Multidisciplinary collaboration<br>• Tools to support communication (e.g., audit and feedback, EHR documentation, huddles, meetings, personnel to coordinate)<br><br>Collective mindfulness was diminished by:<br>• The lack of a reliable process for accurately diagnosing sepsis<br>• Sepsis outcomes that were insufficiently tied to sepsis-related processes<br>• Dynamic priorities<br>• Competing priorities	siloed…" (Quality Physician, Hospital 6)<br><br>"I'm glad that everybody's asking the right question now. Instead of, okay, yeah, quality metrics is not my area, my realm. It is your part. Is your job, not my job. Everybody's taking a part of it." (Hospitalist, Hospital 15)
Organizational knowledge & learning	External recommendations and requirements incentivized hospitals to evolve in their approaches to sepsis care. Evolution in hospitals' approaches to sepsis care was supported by:<br>• Effective use of technology (e.g., EHR)<br>• Interdisciplinary collaboration to interpret data and operationalize improvements in sepsis-care<br>• Provider-directed feedback and education<br>•<br>Hospitals most effectively gained sepsis-related knowledge through a combination of learning approaches: relational (i.e., knowledge-sharing among individuals and groups), didactic (i.e., formal training), and experiential (i.e., engaging with patients with suspected sepsis).	"Knowledge, and just sort of putting that information out to everyone…education probably being 85 percent of what we're doing, and saying it over and over and over again. In multiple forms, right. Email forms, direct information as we're working side-by-side with our team members there. Whatever modality we can." (ED Director, Hospital 14)<br><br>"When there are changes in sepsis care—we have standard meetings…we have a department meeting…Then there's email education if there's a change in practice, there's a change in the electronic form of that practice. Then there's email versions of that education…I think 50 percent of my group or more is in the above 50 age group. Oftentimes, there has to be a little bit of at-elbow support and training for computer stuff that changes just so that those providers are comfortable with that change. Then from the nursing side of things, education gets rolled out in their morning reports and however else they distribute their education."(Physician Administrator, Hospital 17)
Better measurement tools and definitions	Sepsis care quality was measured using imperfect data. Data quality was compromised by:<br>• Frequent changes in sepsis-related performance measures	"[Stroke] and myocardial infarction are discrete entities with gold standard tests and pathologic correlates. That does not exist for sepsis…You can see it, whereas sepsis is a thousand different things." (Nurse Educator, Hospital 11)

	• A lack of valid, reliable data (e.g., inaccurate/missing EHR data)<br>• A lack of training on sepsis-related performance measures<br>• Insufficient engagement of hospital and ED leaders in operationalizing sepsis-related performance measures in clinical practice	"We have an order set. We don't use it. I'll be honest, yeah… We put a lot of work into order sets and make them very burdensome, and then subsequently, nobody uses them." (ED Director, Hospital 19)
System calibration	Coordination of sepsis-related recommendations and requirements across health systems required balancing consistency across hospitals against adaptation to account for individual hospital characteristics (e.g., access to resources).<br><br>Inconsistencies across hospitals contributed variation in performance that was more reflective of individual hospitals' access to resources for complying with sepsis-related recommendations and requirements and optimizing performance data than of actual sepsis care quality.	"Well, they're a tertiary care clinic, so they get a lot of people transferred in… It's a bigger hospital, so there are many more people. A lot of their numbers may be good because they get people transferred in already with a diagnosis of sepsis. There's not any question when they get there what they're supposed to do. ... They got a sepsis coordinator down there. I think, sometimes, maybe there is...a lot of differences in the facilities. ...It's just a lot of things that you try to compare data. It's difficult. Different resources, different facilities, different definitions, different review process." (Physician Administrator, Hospital 18)<br><br>"We use a Vizient data to benchmark against other academic medical centers, and we were nowhere near the top. I mean, there's all these different ways that health systems can be ranked, and we weren't doing a good job." (Sepsis Coordinator, Hospital 20)<br><br>"Our organization continues to get bigger and bigger and then, our partners in the <Market>, for example, adhere to SEP-1. They have been doing that since I think 2016, and they perform really well. This is pure speculation, but perhaps some leaders looked around and say, 'Hey, well, why is the <Market> doing so well with SEP-1? ...Cause we're not focusing on it.' They said, 'Well, maybe you should focus on it.'" (ED Physician, Hospital 2)
Site calibration	Sepsis care quality improved when:<br>• Recommendations and requirements were prioritized throughout health systems<br>• Hospital and ED leaders were engaged in implementing sepsis-related recommendations and requirements	"Resources—any of the community hospitals is gonna come down to resources. When I'm at <academic hospital>, I have a pharmacist that's on my shift with me. That's unheard of at a community hospital in <City> or something. It's awesome, by the way. They actually can look and say what antibiotics they need for sepsis. I go

	• Provider and staff had the resources needed to implement sepsis-related recommendations and requirements (e.g., clinical support and oversight, time to experiment with sepsis-related recommendations and requirements)	and ask them. At <Hospital>, I'm trying to figure this stuff out. I'm looking at—because it matters.” (Medical Director, Hospital 15)
Provider calibration	Compliance with sepsis-related recommendations and requirements was variable due to provider beliefs that metrics did not align with best practices for patient care. To address variation in compliance with sepsis-related recommendations and requirements, hospitals monitored sepsis-related performance data.	“I've heard physicians say, ‘Well, it's not evidence-based, and we don't necessarily agree with it, but we have to implement it and we have to support it because of CMS.’ There's some back-and-forth about doing what's required by CMS and doing what's evidence-based, so there's—I've just heard comments back and forth from a handful of providers about whether they support or believe in the SEP-1 bundle or not.” (ED Physician, Hospital 2)
Technology	• Hospitals used various tools (e.g., Epic dashboard) to support sepsis diagnosis.<br>• The diversity of order sets complicated use.<br>• Healthcare professionals’ perspectives on best practice alerts were mixed: Some found that best practice alerts supported diagnosis, whereas others did not find them to be useful.<br>• EHR system transitions raised concerns about added complexity and potential data loss.<br>• Healthcare professionals expressed a desire for an automated, EHR-embedded sepsis detection tool but noted that the technology is not yet sufficiently sophisticated and challenges obtaining approval for its use.<br>EHR tools’ potential to improve sepsis care was limited by:<br>• Limited relevance to the specific hospitals<br>• Alert fatigue among providers<br>• Discrepancies between tool settings and clinical judgment<br>• A lack of training in how to use the tools<br>• Disruptions related to health system consolidation	“We have an order set. We don’t use it. I’ll be honest, yeah...Well, I’m involved in the EMR groups here for the ER. We have a bad habit, at least as an organization. We put a lot of work into order sets and make them very burdensome, and then subsequently, nobody uses them. I think unfortunately, at least with our what we call the code sepsis order set, it is not an easy order set. I say that from the perspective of it reflects how difficult this measure is to track, and it just includes too much.” (ED Director, Hospital 19)<br><br>“[W]e went from <original EHR> to the new <EHR>, and so the order sets all changed for sepsis.” (ED Director, Hospital 14)<br><br>“[I]f the provider would enter the sepsis diagnosis, it would automatically fire all these BPAs for reminders. If they didn’t put the diagnosis in and if they didn’t start the bundle smart phrase for sepsis, it would never fire. A lot of it wasn’t—we had a lot of processes in place that if a provider or a nurse triggered the key words, it would start that cascade. If they didn’t put the words in, it never cascaded.” (Quality Manager, Hospital 15)
Clinical content	Hospitals sought to bridge the gap between sepsis-related strategies (e.g., SEP-1) and what occurred in clinical practice with strategies such as order sets and training. The efficacy of	“There’s a lot of confusion around sepsis, when to call it sepsis, when not to. I’ve seen a lot of people put sepsis on the chart when there’s no infection, and it just don’t make a lot of sense to me. I’m

	these strategies was limited by (1) the dynamism of both sepsis-related strategies and of clinical practice and (2) the burden of documenting clinically permissible deviations from sepsis-related strategies in practice.	just tryin' to—we're getting better at it. If I understand it, I can teach it, but I don't really fully understand it myself. I don't know." (Physician Administrator, Hospital 18)
Measurement of diagnostic errors	Health systems, hospitals, and EDs measured and monitored sepsis-related performance for the following purposes:<br>• Quality improvement<br>• Compliance with sepsis-related recommendations and requirements<br>• Ensuring payment for care<br><br>These outcomes improved by:<br>• Measuring and monitoring at multiple levels (i.e., system, hospital, and ED)<br>• Leveraging EHR for measurement and monitoring (rather than manually)<br><br>Lags between sepsis-related performance data entry and review (ranging from 6 to 18 months) diminished providers' confidence in performance reports and ability to recall and address concerns.	"Needless to say, the team within CMS that made the decisions to move this forward decided to move it forward, and then, obviously, anything that CMS decides on that has financial implications is a pretty big lever to make sure that we're meeting those requirements." (Physician Administrator, Hospital 2)<br><br>"I think there's more fear in working someone up for sepsis than there's more confidence because of the fear of fluid overload, the fear of injuring kidneys and the fear of providing antibiotics when everyone seems to want antibiotics. I think there is some timidness in that. I think we've really focused on doing the lactic acids and really focus on doing the blood cultures, which we might not get results on blood cultures for three to five days." (Quality Manager, Hospital 15)<br><br>"It's just a lot of things that you try to compare data. It's difficult. Different resources, different facilities, different definitions, different review process." (Physician Administrator, Hospital 18)<br><br>"I sent all misses to the ER Director. At that time, we weren't doing daily abstractions, we were doing CMS. The biggest pushback there, and the biggest issue was, we're looking backwards. That's too far back, and they don't know what happened. They can't do something about what they don't know that happened." (Quality Director, Hospital 18)<br><br>"We get a weekly report every Sunday night…I go through, and I do chart audits and try to see if I can figure out what happened…I think the reason that it's frustrating when I do get feedback is because <Name> is obviously looking in hindsight with 20/20. Most of the misses we have are that we are a busy ER…Now we missed our measure." (Sepsis Coordinator, Hospital 6)

Governance	Hospitals' sepsis-related strategies were developed by health system and hospital leadership and influenced by external organizations that establish sepsis care standards (e.g., CMS).	" We definitely have forums to review new data, literature, practices, and discuss and collaborate with the emergency department of, do we wanna make this change? Do we wanna make changes to our order sets based on it?" (Critical Care Physician, Hospital 7)
Sepsis-related personnel	Sepsis care quality was influenced by a diverse group of stakeholders, including providers in the ED and affiliated departments, nursing staff, ED leadership, sepsis-specific staff (e.g., sepsis coordinators), quality improvement professionals, and pharmacists. These stakeholders' contributions to quality sepsis care were enhanced with more expansive roles and responsibilities (e.g., nurse-driven sepsis protocols) and coordination stakeholders' efforts.	"We used to have a sepsis coordinator. So, it was someone whose job was just to look at charts and do all the auditing. It really falls on people like me who have multiple hats, right? I work clinically. I'm trying to do resident education, medical school and education. Like I said, as a quality, sepsis is just one thing within that wheel. I don't have the bandwidth to review every single sepsis fallout. I just can't do it, right? Similarly, some interested nurses sit on the ED sepsis committee and that, we would love to look at every single fallout and then give a detailed email about what, but it's just so much work. You can't do it." (ED Physician, Hospital 2)
Tasks and Processes	To optimize sepsis care, leaders developed sepsis-related strategies. To operationalize sepsis-related strategies, hospitals often leveraged technologies (e.g., electronic health records, written documents, provider-to-provider messaging, warm handoffs). Sepsis-related technologies' efficacy was sometimes limited by poor implementation (e.g., lack of integration into hospital workflows).	"We have an order set. We don't use it. I'll be honest, yeah...Well, I'm involved in the EMR groups here for the ER. We put a lot of work into order sets and make them very burdensome, and then subsequently, nobody uses them. I think unfortunately, at least with our what we call the code sepsis order set, it is not an easy order set. I say that from the perspective of it reflects how difficult this measure is to track, and it just includes too much." (ED Director, Hospital 19)

Organizational Characteristics: Size	Relative to larger hospitals, smaller hospitals experienced unique challenges to sepsis care quality:<br>• Too few beds to initiate care<br>• Insufficient staff to care for patients<br>• Lack of dedicated sepsis personnel (e.g., sepsis coordinator)<br>• Lower patient volume, limiting opportunities to become familiar with the signs and symptoms of sepsis and procedures for implementing sepsis protocol<br><br>Sepsis care in larger hospitals was compromised by high patient volume.	“The things that went poorly, and those patients were identified as sepsis, we had nowhere to put them. We have 34 beds in our department, not including our super track area…Getting them back to a bed and getting them antibiotics and IVs and everything started was the biggest challenge, just because we didn't have anywhere to put anybody.” (ED Nurse, Hospital 13)<br><br>“Struggles are just sometimes it's staffing related. If we’re short staffed, sometimes it makes it harder for—to get a group in that room to start taking care of the patient. Or versus just the nurse.” (ED Nurse Manager, Hospital 16)<br><br>“Some of the other EDs though that see a lower volume, have less reps and less practice on that, and so that creates, obviously, a disparity in the potential care.” (Physician Administrator, Hospital 2)<br><br>“Sometimes it's also a question of availability of bed space. We get sometimes these patients are in the waiting room. Especially if they've come through the front door and we've done their initial labs. All of a sudden, they don't feel good. We reassess them and their labs have come back up normal. At that point we activate a code sepsis. Sometimes it's a struggle to get an available bed space. We hold a lot of admitted patients down here, um, in the main ER. That's probably one of our biggest challenges.” (ED Nurse Manager, Hospital 16)

Organizational Characteristics: Location	Rural hospitals experienced unique challenges to sepsis care quality:<br>• Patient populations tended to arrive at rural hospitals with more critical health status, prompting providers to more promptly and aggressively treat potential sepsis.<br>• Rural patients were often uninsured, at times compromising early intervention for suspected sepsis.<br>• Rural hospitals tended to be less integrated into their health system, diminishing the relevance of the health system's sepsis-related strategies.<br>• Rural hospitals tended to have lower patient volume, limiting opportunities to become familiar with the signs and symptoms of sepsis and procedures for implementing sepsis protocol.	"We do have a lot of high-acuity patients here, and a lot that come in, but it's also—I think some of it is we're rural, and they wait to come here as a last resort, so they come in sick." (ED Nurse Manager, Hospital 15)<br><br>"Those that don't [have insurance] are less reluctant because of the financial burden of that, so they may present to the ED a lot sicker than others would." (ED Nurse Manager, Hospital 15)
Organizational Characteristics: Type	In contrast to community hospitals and health system spokes, academic hospitals and health system hubs tended to have:<br>• Greater patient volume, increasing the denominator for their sepsis-related performance and providing the opportunity to become familiar with the signs and symptoms of sepsis and procedures for implementing sepsis protocol<br>• More resources to intervene on potential sepsis (e.g., ICUs)<br>• More system strategies related to sepsis care<br><br>Sepsis care quality in academic hospitals and health system hubs was challenged by:<br>• The presence of clinician learners who lacked the time to become proficient in the hospital's sepsis protocol and lacked experience in providing sepsis care<br>• Providers who tended to prioritize clinical judgment over guidelines and sepsis protocols	"I would say a lot of appropriate focus goes to the larger sites, so in <City> market, we have three very high-acuity EDs...that see the vast majority of the sepsis patients. Some of the other EDs though that see a lower volume, have less reps and less practice on that, and so that creates, obviously, a disparity in the potential care...You have to tailor some of the processes in the education to those individual sites a little bit differently to make sure that it's always front of mind." (Physician Administrator, Hospital 2)<br><br>"Resources—any of the community hospitals is gonna come down to resources. Let me explain. When I'm at <academic hospital>, I have a pharmacist that's on my shift with me. That's unheard of at a community hospital in <City> or something. It's awesome, by the way. They actually can look and say what antibiotics they need for sepsis. I go and ask them. At <Hospital>, I'm trying to figure this stuff out." (Physician Administrator, Hospital 2)<br><br>"Because in our department 'cause we're an academic department, we have brand new learners, brand new interns, who may not even be emergency—it might be an ortho intern who's ordering everything." (ED Director, Hospital 11)

Outer Setting (Macro; OTIS)		
External rules and regulations	Recommendations and requirements from the following groups influenced hospitals' sepsis-related strategies:<br>• CMS<br>• Quality benchmarking organizations (e.g., Leapfrog)<br>• Regional leadership<br>• Health system leadership<br><br>The effectiveness of these groups' recommendations and requirements on sepsis care quality was moderated by:<br>• Frequent changes in recommendations and requirements<br>• Conflicts and inconsistencies in the groups' recommendations and requirements<br>• Beliefs that recommendations and requirements do not align with best practices for patient care<br>•<br>Health system performance metrics, pressure from CMS, and national staffing shortages influenced hospitals' sepsis-related strategies.	"At the end of the day, it's tied to reimbursement from government agencies/insurance agencies, so I'm hoping that we still prioritize patient care versus just clicking the boxes and meeting the numbers." (Quality Physician, Hospital 6)<br><br>"I don't know how these CMS regulators come up with this. I feel like they have the best of intentions, but I don't know if they truly understand how it is in practicality and then expect that to be carried out and executed is what we're all scrambling and trying to figure out." (Quality Physician, Hospital 6)<br><br>"Anything that CMS decides on that has financial implications is a pretty big lever to make sure that we're meeting those requirements." (Physician Administrator, Hospital 2)
Interorganizational Relationships	Health system consolidation tended to promote alignment of sepsis-related strategy. Successfully implementing sepsis-related strategy required involving hospital leaders, providers, and staff.	"I think we wanna make sure that overall, we have a similar process, but you are right in the fact that we do have to have some nuances based on the sites and what resources we have." (ED Nurse Manager, Hospital 4)<br><br>"It's pretty independent ...when I talked to them at <community hospital>, it sounds like they're creating their own sepsis committee...I think they are trying to do an independent review as opposed to just, 'Well, we'll just see what the institution says, and we'll do that.' Yes, many things from a leadership standpoint. Initiatives are local." (ICU Physician, Hospital 14)
COVID Impacts	The Covid-19 pandemic influenced sepsis care quality by:<br>• Diminishing providers' ability to comply with sepsis-related recommendations and requirements	"Prior to COVID, we made some grounds with our getting our bundle where it needed to be...but I think with COVID come along, everybody lost focus. It was hard to tell what was COVID, what was

	• Straining baseline resources (e.g., financial, human [i.e., staffing shortages and related loss in sepsis-related knowledge and skills], equipment)<br>• Demoting sepsis as a clinical priority	sepsis. I think it just really threw us all for a loop." (Sepsis Coordinator, Hospital 18)

CMS = Centers for Medicare and Medicaid Services; ED = emergency department; EHR = electronic health record; OTIS = Organization Theory for Implementation Science; TDF = Theoretical Domains Framework; *See Table 4 for hospitals' sepsis-related strategies.
